# Supplementary material for: Asynchronous magnetic resonance elastography: Shear wave speed reconstruction using noise correlation of incoherent waves
Source: Magn Reson Med. 2022 Oct 27;89(3):990–1001. doi: 10.1002/mrm.29502 (PMC9792433; doi:10.1002/mrm.29502)
Supplement: Supplementary file 1 — DATA S1 MATLAB code used to generate the numerical simulations of Figure 1 [file MRM-89-990-s002.zip › k-Wave/helpfiles/example_us_transducer_as_sensor.html]

Using An Ultrasound Transducer As A sensor Example (k-Wave)


# Using An Ultrasound Transducer As A Sensor Example

This example shows how an ultrasound transducer can be used as a detector by substituting a transducer object for the normal `sensor` input structure. It builds on the Defining An Ultrasound Transducer and Simulating Ultrasound Beam Patterns examples.

- Open the file in the MATLAB Editor
- Run the file in MATLAB

## Contents

- Running the simulation
- Forming a scan line

## Running the simulation

In the previous ultrasound examples, the transducer object was used to replace the `source` input of `kspaceFirstOrder3D`. The same transducer object can also be used as a detector by using this to replace the corresponding `sensor` input. An equivalent sensor could be created using a sensor mask containing the grid points for each transducer element, and then averaging the recorded pressure signals over the appropriate grid elements after the simulation is complete. However, directly using the transducer object is both more convenient and more memory efficient (the average pressure across each element is calculated at every time step, and only the average is stored). In this example, two spherical objects embedded within the medium are used as the source, and an ultrasound transducer with 32 active elements is used as the detector. A snapshot is shown below.

The way in which the signals across each element are averaged within k-Wave depends on the setting for `transducer.elevation_focus_distance`. If this is set to `inf`, the signals across the grid points within each sensor element are averaged at each time step and only the average is stored. On the other hand, if an elevation focus is used, a buffer the length of the longest beamforming delay is filled (using a FIFO queue) and the elevation beamforming is computed on the fly once the buffer is filled. In both cases, this significantly reduces the memory requirements compared to storing the complete time history at every grid point within the transducer.

A plot of the returned `sensor_data` is shown below. This is indexed as `sensor_data(element_number, time)`. The signal from the off-axis source that is closer to the transducer is stronger and arrives first.


## Forming a scan line

The raw signals recorded by the transducer can be formed into a scan line by using the functionality of the `kWaveTransducer` class. The `scan_line` method takes the recorded sensor data and forms it into a scan line based on the current `focus_distance` and `receive_apodization` settings.

```
% extract a single scan line from the sensor data using the current
% beamforming settings
scan_line = transducer.scan_line(sensor_data);
```

A plot of the scan line is given below. Even though the signal from the off-axis source is stronger in the individual signals, after beamforming, the signal from the on-axis source is much stronger.
